# Comparison of the impact of two national health and social care integration programmes on emergency hospital admissions

**DOI:** 10.1186/s12913-021-06692-x

**Published:** 2021-07-12

**Authors:** Marcello Morciano, Katherine Checkland, Mary Alison Durand, Matt Sutton, Nicholas Mays

**Affiliations:** 1 Health Organisation, Policy and Economics (HOPE) Research Group, University of Manchester, Manchester, M13 9PL UK; 2 Department of Health Services Research and Policy, Policy Innovation and Evaluation Research Unit, London School of Hygiene & Tropical Medicine, London, WC1H 9SH UK

**Keywords:** Health policy, Social care policy, Integrated care, Vanguard, Pioneers, England

## Abstract

**Background:**

Policy-makers expect that integration of health and social care will improve user and carer experience and reduce avoidable hospital use. [We] evaluate the impact on emergency hospital admissions of two large nationally-initiated service integration programmes in England: the Pioneer (November 2013 to March 2018) and Vanguard (January 2015 to March 2018) programmes. The latter had far greater financial and expert support from central agencies.

**Methods:**

Of the 206 Clinical Commissioning Groups (CCGs) in England, 51(25%) were involved in the Pioneer programme only, 22(11%) were involved in the Vanguard programme only and 13(6%) were involved in both programmes. We used quasi-experimental methods to compare monthly counts of emergency admissions between four groups of CCGs, before and after the introduction of the two programmes.

**Results:**

CCGs involved in the programmes had higher monthly hospital emergency admission rates than non-participants prior to their introduction [7.9 (95% CI:7.8–8.1) versus 7.5 (CI: 7.4–7.6) per 1000 population]. From 2013 to 2018, there was a 12% (95% CI:9.5–13.6%) increase in emergency admissions in CCGs not involved in either programme while emergency admissions in CCGs in the Pioneer and Vanguard programmes increased by 6.4% (95% CI: 3.8–9.0%) and 8.8% (95% CI:4.5–13.1%), respectively. CCGs involved in both initiatives experienced a smaller increase of 3.5% (95% CI:-0.3–7.2%). The slowdown largely occurred in the final year of both programmes.

**Conclusions:**

Health and social care integration programmes can mitigate but not prevent rises in emergency admissions over the longer-term. Greater financial and expert support from national agencies and involvement in multiple integration initiatives can have cumulative effects.

**Supplementary Information:**

The online version contains supplementary material available at 10.1186/s12913-021-06692-x.

## Background

Many health and care systems across the world are trying to introduce better ways of coordinating care, particularly for people with multiple long-term conditions who require help from a range of health and care agencies [1–3]. Better coordinated care is hypothesised to lead to improved patient experience, health gains and more cost-effective provision [4–6]. In England, a systemic challenge to coordinated care lies in the separation between health services (largely fully funded and provided through the National Health Service (NHS)) and adult social care (long-term domiciliary and residential care services, part-funded by local government and means-tested). Successive initiatives have sought to align health and social care better, without going as far as merging the two systems (e.g. [7–10],). Many other countries have separate health and long term care sectors, and face similar challenges.

Despite the several attempts at such integration, there is little evidence that these initiatives have been effective. Their success has often been presented, at least in part, in terms of their ability to reduce the need for emergency hospital admissions [11]. Reducing emergency admission rates has been a feature of English health policy over the past decade and continues to be one of the most commonly used measures of success for system change initiatives [12–14]. While recent initiatives have begun with a range of objectives including improving patient and carer experiences, policy and system objectives have tended to narrow over time leading to a strong emphasis on reductions in emergency admissions [15–19]. To date, however, there has been little evidence of initiatives successfully reducing emergency admissions [20–26]. One reason for the lack of evidence is that the initiatives are short-lived and often overlapping. Another reason is that the different schemes contain different key features, so drawing lessons on the key ingredients of a successful integration initiative is difficult.

In this paper, we assess the impact on emergency admissions of two recent large national integration care initiatives in the NHS in England. We begin by describing their key features and then identify the research questions that the introduction of these schemes enables us to answer.

## Description of the two integrated care programmes

### Integrated care and support Pioneer programme, 2013–18

The Integrated Care and Support Pioneer programme aimed to promote greater horizontal integration between the separate local health and social care systems by encouraging systems to volunteer to develop and implement new ways of working together with the objective of better meeting people’s needs and improving service users’ experience of care [27].

In the first wave of the programme in November 2013, the English Department of Health (DH) (now Department of Health and Social Care (DHSC)) announced 14 Pioneer areas selected from a round of competitive applications, that had been identified as the “most ambitious and visionary” in their plans for health and social care system integration [28]. A second wave of 11 Pioneer areas was subsequently announced in January 2015. Pioneers were either based geographically on single Local Authorities (*n* = 11, e.g. Sheffield or City of Nottingham) or aggregations of lower tier Local Authorities (*n* = 14, e.g. Vale of York or South Devon and Torbay). Each Pioneer was expected to: drive change at “scale and pace”; implement whole system integration including the voluntary sector; adopt a patient-centred perspective based on the “I Statements” developed by National Voices and Think Local Act Personal [29]; and deliver improved patient experience, better patient outcomes and financial efficiencies [28].

Each Pioneer had an account manager from NHS England (NHSE) and had access to networking meetings, an online information platform and a panel of international experts. Pioneers received very limited additional funding in the form of an initial grant of £20,000, later supplemented by a further one-off £90,000 to support development work.

The most commonly reported focus of the Pioneers was on older people with long-term conditions at high risk of hospitalisation involving the establishment of primary care-related community multi-disciplinary teams focused on facilitating hospital discharge or preventing unplanned admissions in this group. All but one of the Pioneers stated in their initial applications that reducing emergency admissions was an aim or an expected outcome of their integration activities [15]. Risk stratification with targeted interventions and preventive strategies to avoid the need for acute hospitalisation were to be employed to achieve this goal. The focus on reducing emergency hospital care use was given even more emphasis by the Pioneers as financial austerity affected local health care budgets after 2013 [15].

### New care model vanguard programme, 2015–18

A new plan for the NHS in England published in 2014, The 5 Year Forward View (FYFV) [30], described a vision for development focussing on new ways of working to improve care delivery rather than structural reform, and aimed to break down barriers between different organisations and care sectors. It was proposed that a number of ‘Vanguards’ would be established to test potential new ways of providing services. In January 2015 [31], local areas (individual organisations or partnerships) were invited to apply to be part of the scheme. The selection of 50 sites was announced in March. Sites were allowed to define their own boundaries and collaborating organisations, but most were associated with one or more Clinical Commissioning Groups (CCGs), responsible for commissioning health care services for defined local populations.

There were two main types of Vanguard: those mainly focused on reducing avoidable hospital admissions and on providing more integrated care out of hospitals; and those focused on the better organisation of care provided by hospitals and emergency services. Within the first group, three different models were designated: multispecialty community providers (MCPs); primary and acute care systems (PACSs); and enhanced care in care homes (ECH) sites. Within the second group, there were urgent and emergency care networks (UECs) and acute care collaborations (ACCs) [31]. These were more hospital-focused initiatives that were managed separately from the other Vanguards [19]. Our analysis focuses upon the first group, as these were community-based programmes which pursued initiatives similar to the Pioneers.

An intensive support programme led by NHSE was established to facilitate the development and spread of new models of care within and beyond the Vanguards, with the following elements [17]:

- Designated national lead for each model;
- Support to develop logic models describing each local scheme;
- 10 support ‘streams’, covering: model design; evaluation and metrics; integrated commissioning and provision; governance, accountability and provider regulation; empowering patients and communities; harnessing technology; workforce redesign; local leadership and delivery; and communications and engagement;
- Local account managers;
- A variety of learning and networking events and opportunities.

The Vanguard programme was also well funded locally, with successful sites eligible for significant amounts of money to support service changes. It is difficult to establish the exact costs of the Vanguard ‘New Care Models’ programme, as some information is not in the public domain and some costs were in the form of secondment of individuals between organisations. However, the National Audit Office [16] estimated that approximately £329 m was given to the Vanguards directly between 2015 and 2018, with an additional £60 m for national support and monitoring (including support staff costs and national and local evaluations).

### Comparing the impact of the Pioneer and vanguard programmes

Hitherto, the Pioneer or Vanguard programmes have been studied separately [15, 19, 32–38], but never in combination. However, a number of Pioneers subsequently applied to become Vanguards, and thus the two programmes overlapped in time and partially in place. It is perhaps no surprise that a proportion of the CCGs in the Vanguard programme had already been involved in the Pioneer programme since prospective Vanguards were required to show that they had already been engaged in system innovation.

In each of the programmes local sites’ plans included the aim of reducing the rate of increase of emergency use of acute hospitals. However, there were some differences in how they proposed to do this. The Pioneers were explicitly focused from inception on improving the links between the NHS and local authority-funded social care, developed their own plans bottom-up with little or no central guidance and received a very modest package of support from NHSE. By contrast, the Vanguards were much more focused on integrating services within the NHS, though not exclusively, developing their local plans within the framework of the five care models proposed by NHSE. Vanguard sites also received a substantial package of support, extra funding for services, and a budget for local monitoring and evaluation. These differences enable the following questions to be addressed:

1. Whether a national system ‘transformation’ programme with extensive implementation and evaluation support performed better in terms of reducing the rate of increase of emergency hospital admissions than a national programme with broadly similar goals but far less support from the centre;
2. Whether a national system ‘transformation’ programme defined in terms of types of ‘new care models’ performed better than a national programme with similar goals but without specific ‘new care models’ defined a priori.
3. Whether sites that had been Pioneers and then Vanguards performed better than sites which had only been involved in one programme or the other.

## Methods

The Pioneers were defined in relation to local authority boundaries, whereas the Vanguards were defined in terms of groups of GP practices. We perform the analysis at the level of NHS Clinical Commissioning Groups (CCGs). CCGs are the statutory bodies with responsibility for commissioning healthcare services for local communities.

A wide range of CCGs across England were involved in either Pioneer or Vanguard programmes, and some CCGs were involved in both. We defined four groups of CCGs: 1) those involved in the Pioneer programme only; 2) those involved in the Vanguard programme only; 3) those involved in both innovative programmes; and 4) those involved in neither. Additional file 1 provides the list of CCGs that formed part of a Pioneer, a Vanguard, or both.

We obtained monthly counts of emergency admissions to all hospitals in England from the Secondary Uses Service (SUS) via NHS England’s national commissioning data repository over a 60-month period covering the 7 months between the announcement of the Pioneer programme and the announcement of the selected sites for wave one (April 2013–November 2013), 14 months before the announcement of both the Vanguard programme and wave two of the Pioneers (November 2013–January 2015) and finally the remaining period of both programmes (to March 2018).

Emergency admissions are defined as those with a ‘specific acute’ treatment function code. Monthly counts were matched to general practices’ registered population counts, made available through NHS Digital and aggregated to CCG level. To account for different population sizes, emergency admissions were expressed as the number per 1000 persons.

We compared average (population unweighted) monthly counts of emergency admissions observed in the four groups of CCGs, before and after the introduction of the two programmes. We smoothed the monthly series by plotting estimates from non-parametric local linear regressions of monthly counts on time (bandwidth 0.6) [39].

We partitioned the study period into five periods: (1) a 7-month period prior to the announcement of the selected wave one Pioneers sites; (2) the subsequent 14-month period when the Pioneer programme had started but the Vanguard programme had not; (3) a 12-month period covering the first year of the Vanguard programme; (4) a 12-month period covering the second year of the Vanguard programme; and (5) and a 15-month period starting in the third year of the Vanguards until the end of both programmes. We then computed population un-weighted group- and period-specific monthly average rates (and 95% confidence intervals, CI) of emergency admission. We tested differences in growth rates across groups by preforming Wald tests [40], using CCGs involved in neither programme as the reference group.

## Results

Table 1 shows the distribution of CCGs between the two programmes (a full list of involved CCGs and a map with their geographical distribution can be found in Additional file 1). Of the 206 CCGs in England in the study period, 86 CCGs had some involvement in the programmes (41.8%). 55% of them were located in the North and Midlands. The two waves of the 25 Pioneers involved 64 CCGs, mainly located in the North and Midlands (58%). Thirty-five CCGs were involved in the Vanguard programme. 70% of them were located in the North and Midlands. Thirteen CCGs were involved in both programmes meaning that, among the 35 CCGs that were Vanguards, 13 (37%) had been part of the Pioneer programme. Ten of them (77%) were located in the North and Midlands. Two of them were involved in both the enhanced care in care home Vanguard initiative and wave two of the Pioneers,

**Table 1:** Clinical Commissioning Groups (CCGs) involved in the Pioneer and/or Vanguard programmes

Type	No. of CCGs	%
Pioneer only	51	24.8
Vanguard only	22	10.7
Involved in both	13	6.3
Involved in neither	120	58.2
Total number of CCGs	206	100

**Fig. 1:**
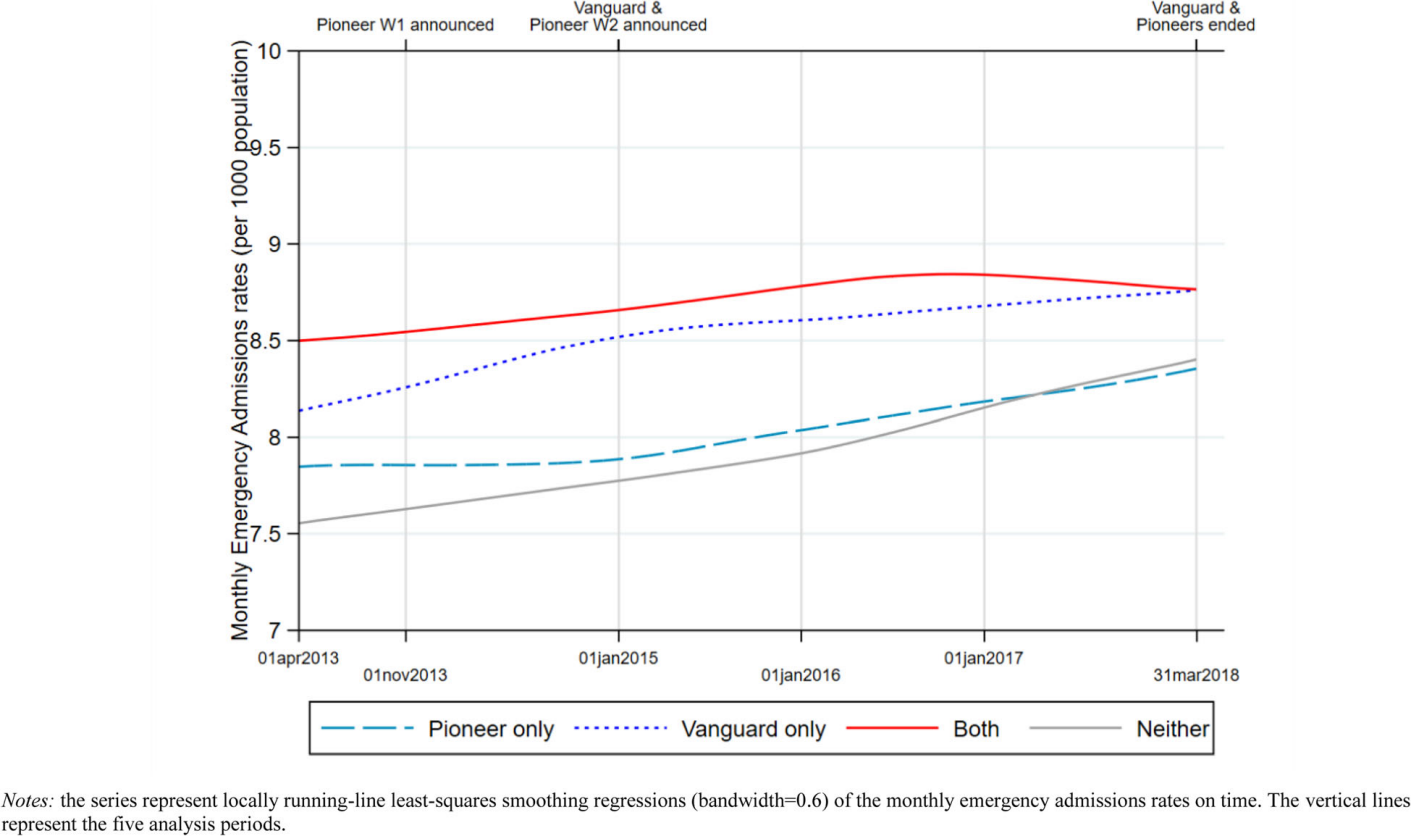
Monthly Emergency admission rates over time and by integration programme in England

Figure 1 reports trends of monthly emergency admission rates observed in the four groups from April 2013 to March 2018.

In April to October 2013, emergency admissions rates were higher in CCGs that were later to take part in the two integration programmes than those which were not. This was particularly the case in CCGs that were later to take part in the Vanguard initiative. From the announcement of the Pioneer wave one (November 2013) until January 2015, emergency admissions rates reduced slightly in those CCGs involved in the programme whereas they increased in the rest of the country. Particularly prominent was the increase in emergency admission rates experienced by CCGs that were later to take part in the Vanguard initiative.

In January 2015, the Vanguard initiative was announced together with the launch of a second wave of Pioneers. The emergency admission rate increased more slowly in Vanguard CCGs than in non-participating CCGs in the first and second years of Vanguard implementation (to December 2017). In the same period, rates of emergency admissions in CCGs that were never Pioneer or Vanguard increased notably, reaching the level experienced by Pioneer wave one CCGs by the end of 2016.

In the third and final year of the programmes, the 13 CCGs involved in the both programmes experienced a reduction in emergency admission rates. This is in contrast with the marked growth observed in the remaining CCGs, particularly the 171 non-Vanguard CCGs. At the end of the period of analysis (March 2018), emergency admission rates observed among the four groups were more similar (within a range of 0.5 emergency admissions per 1000 population) than at the beginning (within a range of 1.0 emergency admissions per 1000 population) (April 2013).

Table 2 reports monthly average rates of emergency admission in the five analysis periods. Rates of emergency admission in Pioneer-only CCGs increased from 7.8 per 1000 in Period 1 to 8.3 per 1000 in Period 5, representing an increase of 6.4% (95% CI: 3.8–9.0%) over the whole period. Over the same period, rates of emergency admissions grew by 11.6% (95% CI: 9.5–13.6%) in CCGs that did not take part in either programme. We can reject the null hypothesis of equal trends at the 1% significance level (Prob > F = 0.0025).

**Table 2:** Average per 1000 emergency admissions rates (and confidence intervals) observed in Pioneer CCGs, Vanguard CCGs and those involved in both and neither programme in the pre- and post-implementation periods

Programme	Periods				
	(1) Pre-Pioneer & pre-Vanguard period (7 months, from Apr 2013 to Nov 2013)	(2) The period with Pioneer wave one only (14 months, from Nov 2013 to Jan 2015)	(3) The first year following the announcement of Vanguard & Pioneer wave two (12 months, from Jan 2015 to Jan 2016)	(4) The second year following the announcement of Vanguard & Pioneer wave two (12 months, from Jan 2016 to Jan 2017)	(5) The last 15 months of the programmes (from Jan 2017 to March 2018)
Pioneer only	7.79 (7.64–7.93)	7.90 (7.79–8.02)	7.90 (7.78–8.02)	8.14 (8.01–8.26)	8.29 (8.17–8.41)
Vanguard only	8.03 (7.86–8.20)	8.46 (8.33–8.59)	8.53 (8.38–8.68)	8.67 (8.51–8.82)	8.72 (8.58–8.87)
Both	8.48 (8.20–8.75)	8.64 (8.41–8.87)	8.63 (8.37–8.89)	8.92 (8.66–9.18)	8.77 (8.55–8.99)
Neither	7.46 (7.37–7.55)	7.76 (7.69–7.84)	7.78 (7.7–7.86)	8.03 (7.94–8.11)	8.33 (8.26–8.40)

*Notes:* population un-weighted group- and period-specific monthly average rates

Rates of emergency admission in Vanguard only CCGs increased from 8.5 per 1000 in Period 2 to 8.7 per 1000 in Period 5, representing an increase of 3.3% (95% CI: 0.3–6.2%). Over the same period, rates of emergency admission in CCGs not involved in either programme grew from 7.8 per 1000 to 8.3 per 1000 (an increase of 7.2%; 95% CI: 5.4–9.0%). However, we cannot reject the null hypothesis of equal trends at conventional statistical levels (Prob > F = 0.1651).

There was a lower rate of increase in emergency admission rates in Pioneer-only CCGs between Periods 1 and 2: emergency admission rates in Pioneer-only CCGs grew from 7.8 per 1000 to 7.9 per 1000 (an increase of 1.5%; 95% CI: − 0.12-3.1%) against an increase from 8.5 per 1000 to 8.6 per 1000 (+ 1.9%, 95% CI: − 0.38 - 4.2) in Pioneer CCGs later involved in the Vanguard programme and an increase from 7.5 per 1000 to 7.8 per 1000 (+ 4.1%; 95% CI: 2.9–5.3) in the CCGs not involved in either programme. Over this period, the growth rate was even higher (+ 5.4%; 95% CI: 3.1–7.7) in CCGs that were later involved in the Vanguard programme but were not participating in the Pioneer programme. Compared to the group of CCGs involved in neither programme, emergency admission rates grew significantly less in Pioneer-only CCGs (Prob > F = 0.0124), whereas the hypothesis of equal trends cannot be rejected at conventional statistical levels comparing to Pioneer CCGs later involved in the Vanguard programme (Prob > F = 0.1084) and CCGs later involved in the Vanguard programme that were not Pioneers (Prob > F = 0.3172).

Between Periods 3 and 5, there was a sharp increase in emergency admission rates in CCGs that took part in neither programme, from 7.8 to 8.3 per 1000, an increase of 7.0% (95% CI: 5.0%–9.0%). Over this period, rates of emergency admissions increased by 2.4% (95% CI: − 0.6% to 5.4%) in Vanguard only CCGs, 1.6% (95% CI: − 1.6% to 4.8%) in CCGs involved in both programmes and 4.9% (95% CI: 3.2% to 6.5%) in Pioneer-only CCGs. Compared with CCGs that did not take part in either programme, the slowdown in the rate of increase in emergency admissions was significant for CCGs involved in the Vanguard programme and the Pioneer programme (Prob > F = 0.0052) and CCGs involved in the Vanguard programme but not involved in the Pioneer programme (Prob > F = 0.0129). Compared with CCGs that did not take part in either programme, the slowdown in the rate of increase in emergency admissions in Vanguard CCGs occurred mainly in the third and final year (period 5) of the programme and for CCGs that had previously been involved in the Pioneer programme. Compared to the previous year (period 4), emergency admission rates of CCGs involved in both programmes reduced from 8.9 to 8.8 per 1000 (a reduction of 1.7%, 95% CI: − 4.4% to 0.1%) compared to an increase of 3.7% (95% CI: 1.7% to 5.8%) observed in CCGs that did not take part in either programme.

## Discussion

Integrated care initiatives are seen internationally as an important mechanism in managing healthcare for an aging population [1–3]. It is commonly claimed in the UK and elsewhere that improving integration will lead to the more effective care of people in the community, thus reducing the use of expensive hospital care and improving system efficiency. However, despite the widespread support and considerable energy devoted by policymakers and care professionals to better integration of health and social care, there is little evidence that these initiatives are successful in reducing the rise in emergency hospital admissions. This is partly due to the heterogeneity of these integration initiatives and partly due to their overlapping introduction. We took advantage of national data and the phased nature of two major national integration programmes in England to investigate whether these programmes impacted on emergency hospital admission rates.

Compared with the areas that did not participate in either programme, CCGs that took part only in the Pioneer programme experienced a stabilisation of emergency admission rates after the announcement of the programme in November 2013, before rates rose again from 2015 in almost exactly the same manner as rates in the CCGs that took part in neither programme. Emergency admission rates grew less in CCGs involved in the Vanguard programme when this programme was introduced but the rates still remained higher than other CCGs. However, emergency admission rates grew less over time in the CCGs involved in both integration programmes. Their emergency admission rates declined from the end of the second year of the programmes, moving towards the levels seen in the other CCGs.

Previous separate analyses of the two integration programmes appeared to show a limited and temporary association between first wave Pioneer status and a lower rate of increase in emergency hospital admissions [32] and a modest reduction in the rate of increase of emergency admissions among Vanguards compared to non-Vanguards. The latter took time to develop, being seen mainly in the last year of the programme [37]. The Vanguard analysis also highlighted the variation in changes in admission rates among Vanguards. It is not clear why some sites succeeded in reducing the rate of increase of emergency admissions and others did not.

The findings of the current analysis shed further light on these previous findings. The current analysis shows that the overall rise in the rate of emergency admissions observed in England, especially in 2017/2018, was partially offset by reductions in sites involved in both Vanguard and Pioneer initiatives. These reductions occurred mainly in the three CCGs that were wave one Pioneers and also later took part in the Vanguard programme (Canterbury and Coastal, Tower Hamlets and West Cheshire CCGs) and in two CCGs involved in the concomitant Vanguard and wave two Pioneer programmes (Nottingham City and Wakefield CCGs). Of particular interest are the two sites that were involved in both the enhanced care in care home Vanguard initiative [41] and wave two of the Pioneers. The enhanced medical care to care homes initiative is a very specific intervention with a plausible direct mechanism of action to reduce emergency use of hospitals. This initiative did not figure in the Pioneer programme. Continuous involvement over time in successive national integration initiatives with some focus on reducing hospital admissions appears to have strengthened the ability of these CCGs to prevent the rise in emergency admissions observed across other parts of England, especially in 2017/2018. Overall, this suggests that integration initiatives may be additive – i.e. that reducing unplanned emergency admissions to hospital takes sustained effort across a health and care system over a prolonged period of time. In addition, it seems that that such effects are likely to be enhanced by interventions such as enhanced medical care in care homes which are very directly targeted at people who are at high risk of hospitalisation.

Whilst the Vanguard and Pioneer programmes both had similar aims – to improve the integration of care between health and social care and to improve care in the community so as to reduce unplanned admissions to hospital – there were significant differences between the programmes. Most notably, the Vanguard programme was better supported both financially and in terms of investment in support functions at national level. As such, it might have been expected that Vanguard outcomes would have been significantly better than those associated with Pioneers. Our analysis shows a modest advantage for Vanguard sites in that their reduction in the growth in emergency admissions was better sustained. However, the costs associated with the Vanguard programme were much greater. In addition, the Pioneer programme was more permissive, in that local areas were encouraged to design solutions fitted to their local context, whereas the Vanguard programme required applicants to define their approach according to a set of broad system models. Our analysis does not clearly show any advantage associated with either a bottom-up or a more structured, top-down initiative design.

This analysis has important limitations. First, we were not able to account for local contextual factors which might have affected the adoption and implementation of integration processes. Moreover, implemented initiatives were heterogeneous across sites and difficult to describe in detail using interviews and documentary analysis [15, 19, 32–37]. A recent evidence synthesis of 115 local Vanguard evaluation reports concluded that none of the reports offered “*explanations and/or nuanced insights into the Vanguard operation*” [38], making cross-initiatives comparisons difficult. Second, our method does not rule out the possibility that confounding events, unrelated to the integration processes, may have differentially affected the different groups of CCGs being compared in the post-implementation period. These are hard to account for without a difference-in-difference analysis with multiple groups. However, such an approach relies upon the ‘parallel trends’ assumption which is clearly not satisfied by the graphical inspection of the pre-Pioneer and pre-Vanguard trends in admission rates plotted in Fig. 1. Since there was a tendency for CCGs involved in the two programmes to have higher starting emergency admission rates, there is always a possibility that the analysis is vulnerable to the problem of ‘regression to the mean’ [42] and that changes observed are not all the direct result of integration initiatives. Third, our analysis defines periods (of unbalanced length) in relation to the official inception dates of programmes. This may not represent the precise ‘start’ of each programme, given that the localities were almost certainly attempting to make their care more integrated before entering the programmes and did not necessarily begin their new integration schemes precisely when the programmes were officially announced. Indeed, it seems likely that there was a variable, hard-to-discern lag between the two events. Finally, our analysis assesses differences in admission rates at the level of CCGs. However, the functional and geographical borders of the sites involved in the Pioneers and Vanguard programmes did not map precisely onto individual CCGs in all cases. This is because the Pioneer programme geographies were defined predominantly by local authority areas whereas the Vanguards were organised by CCG. This means that we might have underestimated the effect of on hospital admissions where the Pioneers and Vanguard initiatives did not fully align with the CCG boundaries.

Despite these limitations, a strength of the current analysis is the ability to look at the cumulative effect of participation in successive high profile national integration programmes in England, given that it is often argued that such programmes are not implemented for long enough to demonstrate impacts. To our knowledge this is the first such analysis in England.

## Conclusion

Over the past decade, there has been international interest in health and care integration policies aimed at the simultaneous improvement of patient experience and health status while reducing the cost of health care [6, 43]. This paper provides an original, longer term, analysis of the impact of the two largest national integrated care initiatives in England on emergency hospital admissions. While it is neither possible to attribute causality to the relationships identified, nor to identify the precise drivers within sites of the trends observed, the findings do suggest that integration initiatives at the system level need to be sustained over time to have any chance of changing use of hospital care. By contrast, nationally led policy initiatives in the NHS tend to take place in rapid succession and over short periods of time [44, 45]. The Vanguard programme, for example, was limited to 3 years of funding, with funding for the second and third years contingent upon success in improving a number of metrics, including those for emergency admissions [19]. The Pioneer programme was unusual in having a just over 4 year lifespan, at least for wave one sites, though it was rapidly ‘overtaken’ by the far better resourced Vanguard programme. The current analysis suggests that expecting programmes to deliver major systemic change over such short timescales is probably unrealistic. Improving health system performance by improving integration between the health and long term care sectors is an extended project, in which progress is likely to be incremental rather than ‘transformational’ [30]. This has significant implications for future support and guidance for integration projects, suggesting, for example, that support may need to be sustained over longer periods of time. This will be tested further in an ongoing analysis of changes in outcomes following the termination of the Vanguard programme.

Finally, the findings indicate that it would be worthwhile to undertake more detailed qualitative research in the small number of seemingly successful areas in both the Vanguard and Pioneer programmes to identify how they achieved their changes in emergency hospital use. Such research could attempt to tease out from interviews with frontline and managerial staff the precise range and nature of the organisational and service changes that had been put in place in these local health and care systems and the time frames over which they had been implemented, along with information about the populations targeted by specific integration initiatives, together with an indication of the quality of local leadership, human and technical resources and local participants’ own explanations of how these changes were able to change patterns of emergency hospital use.

## Supplementary Information

**Additional file 1: Table S1.** List of Clinical Commissioning Groups participating in the Pioneer (63) and Vanguard (35) integration programmes. **Figure S1.** Location of Clinical Commissioning Groups participating in the Pioneer and/or Vanguard programmes.

## Data Availability

Aggregate, non-identifiable data used were taken from the Secondary Uses Service via NHS England’s national commissioning data repository and provided by NHS England. Neither the funders nor the collectors/curators/delivers of the data bears any responsibility for the analyses or interpretations presented here. The datasets generated and analysed during the current study are not publicly available but can be requested through NHS England. All analyses were performed in STATA/MP 14.2. The statistical code is available from the corresponding author upon request.
