# Supplementary material for: Comparison of the impact of two national health and social care integration programmes on emergency hospital admissions
Source: BMC Health Serv Res. 2021 Jul 12;21:687. doi: 10.1186/s12913-021-06692-x (PMC8274044; doi:10.1186/s12913-021-06692-x)

**Appendix 1:**

Table S1: List of Clinical Commissioning Groups participating in the Pioneer (63) and Vanguard (35) integration programmes

| CCG (in alphabetical order) | Pioneer | Vanguard |
| --- | --- | --- |
| Airedale, Wharfedale And Craven | X |  |
| Ashford | X |  |
| Barnsley | X |  |
| Birmingham Crosscity |  | X |
| Birmingham South And Central |  | X |
| Blackpool | X | X |
| Bolton | X |  |
| Brent | X |  |
| Bury | X |  |
| Calderdale |  | X |
| Cambridgeshire And Peterborough |  | X |
| Camden | X |  |
| Cannock Chase | X |  |
| Canterbury And Coastal | X | X |
| Central London (Westminster) | X |  |
| Corby |  | X |
| Cumbria |  | X |
| Dartford, Gravesham And Swanley | X |  |
| Dudley |  | X |
| Ealing | X |  |
| East And North Hertfordshire |  | X |
| East Lancashire |  | X |
| Eastern Cheshire | X |  |
| Erewash |  | X |
| Fareham And Gosport |  | X |
| Fylde & Wyre | X | X |
| Greenwich | X |  |
| Hammersmith And Fulham | X |  |
| Harrogate And Rural District |  | X |
| Harrow |  |  |
| Heywood, Middleton And Rochdale | X |  |
| Hillingdon | X |  |
| Hounslow | X |  |
| Isle Of Wight |  | X |
| Islington | X |  |
| Kernow | X |  |
| Lancashire North |  | X |
| Leeds North | X |  |
| Leeds South And East | X |  |
| Leeds West | X |  |
| Manchester | X |  |
| Mansfield And Ashfield | X | X |
| Nene |  | X |
| Newark & Sherwood | X | X |
| Newcastle Gateshead |  | X |
| Newham | X |  |
| North East Hampshire And Farnham |  | X |
| North Staffordshire | X |  |
| Northumberland |  | X |
| Nottingham City | X | X |
| Nottingham North And East | X |  |
| Nottingham West | X |  |
| Oldham | X |  |
| Redditch And Bromsgrove | X |  |
| Rushcliffe | X | X |
| Salford | X | X |
| Sandwell And West Birmingham |  | X |
| Sheffield | X |  |
| Somerset | X | X |
| South Cheshire | X |  |
| South Devon And Torbay | X |  |
| South Kent Coast | X |  |
| South Lincolnshire |  | X |
| South Tyneside | X |  |
| South Worcestershire | X |  |
| Southend | X |  |
| Stafford And Surrounds | X |  |
| Stockport | X | X |
| Stoke On Trent | X |  |
| Sutton |  | X |
| Swale | X |  |
| Tameside And Glossop | X |  |
| Thanet | X |  |
| Tower Hamlets | X | X |
| Trafford | X |  |
| Vale Of York | X |  |
| Vale Royal | X |  |
| Wakefield | X | X |
| Waltham Forest | X |  |
| West Cheshire | X | X |
| West Kent | X |  |
| West London | X |  |
| West Norfolk | X |  |
| Wigan Borough | X |  |
| Wirral |  | X |
| Wyre Forest | X |  |

*Notes:* 86 CCGs (not shown) did not participate in either programme.

Figure S1: Location of Clinical Commissioning Groups participating in the Pioneer and/or Vanguard programmes


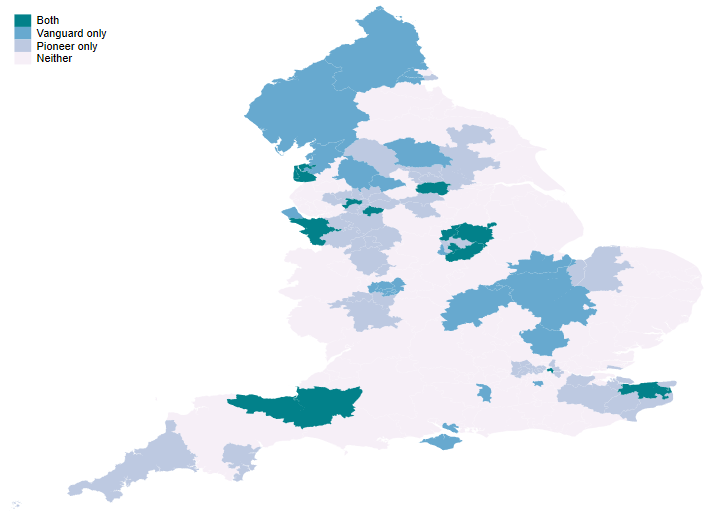

Supplement: Supplementary file 1 — Additional file 1: Table S1. List of Clinical Commissioning Groups participating in the Pioneer (63) and Vanguard (35) integration programmes. Figure S1. Location of Clinical Commissioning Groups participating in the Pioneer and/or Vanguard programmes. [file 12913_2021_6692_MOESM1_ESM.docx]
